# Association of Nrf2 Single Nucleotide Polymorphism rs35652124 and FABP4 Levels with Peripheral Artery Disease Among Type 2 Diabetes Mellitus Pakistani Population

**DOI:** 10.3390/cimb47070530

**Published:** 2025-07-09

**Authors:** Iqra Ayaz, Nakhshab Choudhry, Amna Ihsan, Tehreem Zubair, Aamir Jamal Gondal, Nighat Yasmin

**Affiliations:** 1 Department of Pathology, King Edward Medical University, Lahore 54000, Pakistan; iqra75650@gmail.com; 2 Department of Biochemistry, King Edward Medical University, Lahore 54000, Pakistan; drnbchoudhry@gmail.com (N.C.); dramnaihsan@gamil.com (A.I.); 3 Department of Medicine, Al-Aleem Medical College, Lahore 54600, Pakistan; tehreemzubair01@gmail.com; 4 Department of Biomedical Sciences, King Edward Medical University, Lahore 54000, Pakistan; ajgondal119@gmail.com

**Keywords:** peripheral arterial disease, type-2 diabetes mellitus, FABP4, Nrf2, Pakistan

## Abstract

Peripheral arterial disease (PAD) is a macrovascular diabetic complication, characterized by atherosclerotic plaque formation due to hyperglycemia and dyslipidemia. The molecular mechanisms involved in PAD-T2DM pathogenesis will help in understanding and early prognosis; therefore, we aim to evaluate FABP4 levels and Nrf2 single-nucleotide polymorphisms (SNPs) among PAD-T2DM patients. In a case-control study, 123 samples (healthy control HC, T2DM, and PAD-T2DM; *n* = 41 each) were collected from the diabetic foot clinic at Mayo Hospital, Lahore. Baseline and biochemical data were collected. PAD diagnosis was established by measuring the ankle-brachial index with color Doppler ultrasound. Serum FABP4 levels were measured using an ELISA. Nrf2 SNP rs35652124 analysis was performed by restriction fragment length polymorphism. PAD-T2DM prevalence was higher among male subjects (61.1%). Fasting plasma glucose levels (*p* = 0.02), total cholesterol (*p* < 0.0001), and LDL-cholesterol (*p* = 0.01) were significantly higher in PAD-T2DM as compared to T2DM. SNP association analysis showed that homozygous genotype TT (OR: 3.85, 95% (CI): 1.22–12.11, *p* = 0.02) and T-allele (OR: 1.31, 95% (CI): 1.31–4.67, *p* = 0.005) were significantly associated with PAD-T2DM. FABP4 levels were higher in the PAD-T2DM group as compared to T2DM (*p* < 0.0001) and were significantly associated with Nrf2 SNP genotype TT (*p* < 0.001) and CT (*p* = 0.01) in PAD-T2DM. Our results showed, for the first time, that the Nrf2 SNP is significantly associated with PAD-T2DM and FABP4 levels compared to T2DM.

## 1. Introduction

Type 2 diabetes mellitus (T2DM) is a critical non-communicable, chronic metabolic disease, primarily characterized by the presence of insulin resistance due to the gradual loss of insulin secretion from islet β-cells [1]. The prevalence rate of diabetes has increased from 3.2% in 1990 to 6.8% in 2021. About 96% of all diabetes cases are T2DM, with more than 80% diabetic population belonging to low-income countries. T2DM is a multifactorial polygenic disease affected by several environmental and genetic risk factors. Obesity has been considered a major risk factor for T2DM. Ectopically built-up deposits of fat, accompanied by inflammation and oxidative stress, are considered the primary reasons for the T2DM onset and progression, along with other metabolic derangements. On the other hand, familial incidence is estimated to be 30–70%, which largely depends on the age of T2DM onset and glycemic index [2,3,4]. The perpetual hyperglycemic condition resulted in endothelial dysfunction, specifically targeting organ damage and enhancing the risk of both microvascular and macrovascular complications [5].

Peripheral arterial disease (PAD), one of the macrovascular complications of T2DM, arises more frequently among T2DM patients [6]. Atherosclerosis is the main pathological process for the induction of PAD due to the macrophage functional dysregulation, complemented with cellular injury due to hyperglycemia and dyslipidemia [3,7,8]. This compromised state of the cell leads to chronic inflammation and atrial fibrillation, resulting in a decline in vascular function [9,10]. PAD is characterized by the arterial constriction of the lower limb due to the atherosclerotic occlusion [11,12]. Despite being a debilitating disease, PAD remained undertreated compared to other atherosclerotic disorders due to the lack of awareness [13,14,15]. PAD is recognized as the third dominant cause of atherosclerosis-related morbidity and mortality, with over 200 million people affected globally, and 172 million of whom belong to low- and middle-income countries [16,17,18]. Clinically, high variability has been found during PAD diagnosis; either asymptomatic frequent or intermittent claudication is reported, while acute limb ischemia may occur at an advanced stage [17,19]. Higher incidence of PAD and T2DM comorbidity (20–30%) enhanced the risk of coronary artery disease, amputation of the lower limb, and ultimate demise. The complex pathophysiological relationship between PAD and T2DM highlights the importance of PAD-T2DM diagnosis at an early stage for timely intervention and management [12,20,21,22].

Fatty acid binding protein 4 (FABP4) is predominantly expressed in macrophages and adipocytes and is released as an adipokine into circulation, aligning cholesterol trafficking, inflammation, and oxidative stress [22,23,24,25]. FABP4’s role in atherosclerosis is evident from its elevated expression in macrophages during intracellular lipid accumulation and atherosclerotic plaque formation [26,27,28,29,30]. Contrarily, the formation of foam cells in macrophages was impaired by the suppression of FABP4 [31]. FABP4 levels have been found to be higher and associated with T2DM risk and PAD-T2DM in previous studies [32,33,34]. Several other cellular outcomes are observed due to the exogenous expression of FABP4, including the accelerated production of hepatic glucose, glycolysis obstruction, limited utilization of glucose by muscles, insulin secretion by β-cells, inhibition of glucose oxidation, and insulin signaling pathway [24,35,36,37,38,39].

Due to the saturation of glycolysis, uncontrolled oxidative stress is induced by suppressing erythroid 2-related factor 2 (Nrf2) signaling [5,40,41]. Nrf2, an antioxidant regulator, counteracts oxidative stress and is essential for the differentiation of adipocytes [42]. Nrf2 is significantly involved in atherosclerosis-induced inflammatory responses, such as Nrf2 inactivation in macrophages, which results in increased uptake of low-density lipoproteins and enhanced expression of inflammatory cytokines [43,44,45]. Under normal conditions, Nrf2 regulates oxidative stress, thus protecting pancreatic β cells and maintaining glucose homeostasis. Nrf2 activation enhanced the transcriptional regulation of muscle cells, leading to improved glucose uptake and tolerance [46,47,48]. It was observed that Nrf2 activation for a shorter time period results in improved insulin resistance status, while uninterrupted activation leads to impaired insulin signaling due to the excessive removal of reactive oxygen species [42]. Nrf2 SNP rs35652124 is located in the promoter region of the Nrf2 gene. It may affect the binding site of Nrf2 myeloid zinc finger 1 (MZF1). As a result, the regulation of Nrf2 transcriptional activity was reduced [13]. The reduced Nrf2 activity leads to oxidative stress, which supports the cardiovascular risk development in the presence of obesity, diabetes mellitus, and atherosclerosis [49].

Keeping in view the Nrf2 and FABP4 independent roles among diabetes, only a few reports are available about the Nrf2 and FABP4 roles among PAD-T2DM patients. Nrf2 genetic deletion leads to the lower expression of FABP4 and CD36, suggesting Nrf2’s role in the accumulation of tubular lipids through FABP4 and CD36. However, FABP4 transcriptional regulation through Nrf2 signaling needs to be addressed [50]. Due to the lack of data and a unique genetic framework, we aimed to address whether single-nucleotide polymorphisms (SNPs) of Nrf2 are associated with PAD-T2DM patients and FABP4 levels among the Pakistani population.

## 2. Materials and Methods

### 2.1. Study Design and Setting

This study was conducted as a case-control study at the Department of Biomedical Sciences, Advanced Research Center for Biomedical Sciences (ARCBS), King Edward Medical University. The study was approved by the Advanced Study and Research Board, vide No. 123/KEMU/2024, dated 2 January 2024. Informed consents were obtained from all study participants.

### 2.2. Sample Collection

A total of 123 blood samples, including healthy controls (HC, *n* = 41), T2DM (*n* = 41), and PAD-T2DM (*n* = 41), were collected from the diabetic foot clinic at Mayo Hospital, Lahore, Pakistan, between January 2024 and June 2024. A non-probability convenient sampling technique was used for sample collection. HCs were selected without diabetes or any systemic inflammatory disease. Patients with acute limb ischemia and a history of acute coronary syndrome were excluded. Diabetes diagnostic criteria were established based on HbA1c levels (non-diabetic: 5.7%, pre-diabetic: 5.7–6.4%, and diabetic: ≥6.5%) and fasting plasma glucose levels of ≥126 mg/dL for diabetes and 100–125 mg/dL for pre-diabetes with at least 8 h of fasting [51,52]. Baseline and biochemical data were collected, such as gender, age, BMI, LFTs, RFTs, cholesterol levels, and electrolyte concentration measurements.

### 2.3. Lower Extremity Arterial Duplex Ultrasonography

PAD diagnosis was established by measuring ankle brachial index (ABI; ≤0.90 value for PAD) with color Doppler ultrasound (MyLabEight Exp, Esaote, Italy). The same radiologist performed all the Doppler analyses by measuring the systolic blood pressure of both arms and ankles with the patient lying in a supine position as described previously [53]. Briefly, the individual was in a supine position with a slight outward rotation of the hip and leg, with a probe placed in their groin. The scan began at the inguinal crease, with the transducer placement horizontal over the common femoral artery (CFA) by locating the “Mickey Mouse” view. CFA was examined in the longitudinal view by turning the probe 90° clockwise. In the longitudinal view, plaque and aneurysms on two-dimensional and color modes were checked. Profunda femoris artery (PFA) and superficial femoral artery (SFA) were examined by evaluating the PFA off of the CFA bifurcation, and then the proximal, middle, and distal SFA. Finally, the examination of the popliteal artery is performed with the knee in flexion. The probe was placed behind the knee, and the entire popliteal artery behind the knee was evaluated by moving the probe down the calf until the vessels bifurcate. The degree of stenosis was assessed by velocity ratio measurements. The peak systolic velocity ratio between the stenotic lesion and the unaffected section of the vessel is used to calculate this ratio. A ratio of <1.5 indicates normal, 1.5 to 2 indicates 30% to 49% stenosis, ratios of 2 to 4 and above 4 indicate 50% to 75% and over 75% stenosis, respectively, while no flow indicates an occluded vessel.

### 2.4. Enzyme-Linked Immunosorbent Assay (ELISA)

Serum FABP4 levels were measured using the FABP4 ELISA kit (cat # EH177RB, Thermo Fischer Scientific, Waltham, MA, USA) as per the kit instructions. The analytical sensitivity of the kit was 38 pg/mL, and the assay range was 38–9000 pg/mL. All reagents were prepared according to the kit protocol. Serial dilutions of the standards were performed to obtain the standard curve. After adding all samples and standards, the ELISA microplate was incubated for 2 h at room temperature. Then, the biotin-conjugate was added to the samples, and the plate was incubated for another hour with gentle shaking. The anti-human FABP4 antibody was coated with streptavidin-horseradish peroxidase (HRP), followed by a second incubation for 1 h with gentle shaking. The microplate was washed three times for the removal of the unbound HRP using an ELISA microplate washer (Fisher Scientific ACCU Wash Versa Microplate Washer, model # 14377578, Thermo Fischer Scientific, Waltham, MA, USA). The microplate wells were filled again with the substrate solution reactive with HRP. The plate was incubated for 30 min after the addition of TMB substrate in the dark. Gently tapped the plate after the addition of the stop solution. Color in the plate wells was developed. It was observed by taking absorbance at 450 nm using the ELISA Reader (Multiskan FC Microplate Reader, Model # 14-377-576, Thermo Fischer Scientific, USA). The value of optical density was directly related to the concentration of human FABP4 levels. The serum levels of the human FABP4 in the sample were measured using the standard curve.

### 2.5. Extraction of DNA from Blood and Nrf2 SNP Detection by Restriction Fragment Length Polymorphism

Total genomic DNA was isolated from blood using the GeneJET Whole Blood Genomic DNA Purification Kit (catalogue # K0512, Thermo Fischer Scientific). Nrf2 SNP rs35652124 was analyzed by restriction fragment length polymorphism. PCR amplification was performed using the following primers: forward 5′-CCT TGC CCT GCT TTT ATC TC-3′ and reverse 5′-CTT CTC CGT TTG CCT TTG AC-3′. Sterile nuclease-free water was used as the negative control. PCR products were purified using the GeneJET PCR Purification Kit (catalog # K0701, Thermo Fischer Scientific). The purified PCR product (264 bp) was restricted using BseRI (Thermo Fischer Scientific, Waltham, MS, USA). The restriction product was run on a 3% agarose gel with genotype products of CC (264 bp), CT (264 bp, 192 bp, and 72 bp), and TT (192 bp and 72 bp) [54]. Statistical analysis was performed using SPSS version 26.0. Continuous variable data were presented as mean ± standard deviation (SD). Genotype frequency and distribution were analyzed by the chi-square test and the Odds Ratio. The *p*-values of <0.05 were considered statistically significant.

## 3. Results

### 3.1. Clinical Characteristics of the Study Groups

Of the total, 69/123 (56.1%) samples were collected from males and 54/123 (43.9%) from females. The gender-wise distribution of samples among the three groups revealed that male subjects with PAD-T2DM were higher (25/41, 61.1%) compared to those with T2DM and healthy controls (HC; 22/41, 53.7%). On the other hand, female subjects between T2DM and HC were higher (19/41, 46.3%) as compared to PAD-T2DM (16/41, 38.9%). Fasting plasma glucose levels (*p* = 0.02), total cholesterol (*p* < 0.0001), and LDL-cholesterol (*p* = 0.01) were significantly higher among the PAD-T2DM group as compared to the T2DM group. The detailed results of the clinical characteristics of patients are given in Table 1.

### 3.2. Association and Genotype Distribution Analysis of Nrf2 SNP rs35652124

Nrf2 SNP rs35652124 genotypic analysis showed that the polymorphic genotype TT is more frequent in the PAD-T2DM group (53.7%) as compared to the T2DM (24.4%) and HC (39.0%) groups, while higher frequency of heterozygous CT genotype was found among the T2DM (41.5%) as compared to the PAD-T2DM (26.8%) and HC (17.1%). Association analysis revealed that homozygous genotype TT (OR: 3.85, 95% (CI): 1.22–12.11, *p* = 0.02) and T allele (OR: 1.31, 95% (CI): 1.31–4.67, *p* = 0.005) were significantly associated with the PAD-T2DM as compared to the T2DM. The details of SNP genotype and frequency distribution are given in Table 2.

### 3.3. Measurement of FABP4 Levels Among Study Groups

Circulatory FABP4 levels were higher among the PAD-T2DM group as compared to the T2DM and HC groups. The results were statistically significant among each other (*p* < 0.0001, each). The results are presented in Table 3.

Furthermore, we correlated the plasma FABP4 levels with clinical parameters. Pearson correlation coefficient analysis showed weak-positive correlation of HbA1c (*r* = 0.0371, *p* = 0.81), FPG (*r* = 0.3574, *p* = 0.02), BMI (*r* = 0.1728, *p* = 0.27), total cholesterol (*r* = 0.2141, *p* = 0.17), and LDL-cholesterol (*r* = 0.0791, *p* = 0.62) with PAD-T2DM. We analyzed the distribution of FABP4 serum levels among Nrf2 SNP genotypes. It was observed that FABP4 levels were significantly associated with Nrf2 SNP genotypes TT (*p* < 0.001) and CT (*p* = 0.01) in PAD-T2DM as compared to T2DM. The results are given in Table 4.

## 4. Discussion

Metabolic dysfunction, persistent glycemic and oxidative stress result in low-grade chronic inflammation and tissue vasculature impairment, leading to the development of PAD among T2DM patients [22]. Hence, scarce data about PAD-T2DM from Pakistan is available. Therefore, we aimed to investigate the association of FABP4 levels and Nrf2 SNPs in PAD-T2DM patients from Pakistan. Our results demonstrated that PAD-T2DM is more prevalent among male (61.1%) subjects. Contrarily, the higher prevalence of PAD-T2DM among females has been reported globally [55], including Pakistan (65.6%) [56]. However, regardless of gender, the prevalence of PAD-T2DM is higher among the Pakistani population, as evidenced by several studies, such as 19.9% [57], 28.5% [58], 31.6% [49], 39.28% [59], and 41% [60]. We observed that fasting plasma glucose levels, total cholesterol, and LDL-cholesterol were significantly associated with the PAD-T2DM group compared to the T2DM group. In agreement with our results, other reports have suggested that obesity and hypercholesterolemia are associated with PAD-T2DM [56,57,61]. The deposits of lipids and inflammatory arterial cells progressively lead to atherosclerotic plaque formation, resulting in PAD development [62].

Cellular oxidative stress induced as a result of hyperglycemia and dyslipidemia causes irreversible damage to the vascular system in T2DM patients [63]. Moreover, oxidative stress has been found to be associated with the complications of T2DM [64]. Therefore, the study of oxidative stress-related molecules may give insight into the molecular mechanisms of PAD-T2DM. In the present study, we dissected the genetic role of Nrf2 in the development of PAD-T2DM. Our results showed that the Nrf2 SNP rs35652124 polymorphic genotype TT was more frequent in PAD-T2DM (53.7%) as compared to T2DM (24.4%). Genotype association analysis showed that the homozygous genotype TT (*p* = 0.02) and the T allele (*p* = 0.005) were significantly associated with PAD-T2DM as compared to T2DM. Only a few reports have shown an association between the Nrf2 SNP rs35652124 TT genotype and the T allele and T2DM, with or without diabetic foot ulcer [65,66]. However, no data on the Nrf2 SNP rs35652124 in PAD-T2DM are available globally, including from Pakistan. Therefore, our study is the first to report the association of Nrf2 SNP rs35652124 with PAD-T2DM.

It has been documented that obesity and oxidative stress are associated with T2DM through the abnormal production of adipokines [67]. FABP4 is an adipokine released into circulation. It is reported previously that higher FABP4 plasma levels are associated with exacerbated PAD conditions [68]. It was reported that FABP4 levels were higher in T2DM patients [25,69,70]. We measured circulatory FABP4 levels in PAD-T2DM. Our results showed that FABP4 levels were significantly higher among PAD-T2DM (25.22 ng/mL) as compared to T2DM (18.69 ng/mL) and HC (9.45 ng/mL). Only a few reports have reported FABP4 levels in PAD-T2DM patients, such as lower FABP4 levels in T2DM (10.3 ± 7.59 ng/mL) compared to PAD-T2DM (23.34 ± 15.27 ng/mL), although no healthy controls were included in the study [22]. Our observation suggested that FABP4 levels among T2DM are already higher compared to healthy controls, and further enhancement is observed among PAD-T2DM patients. This may indicate that the duration of T2DM affects the circulatory levels of FABP4. With prolonged T2DM incidence, FABP4 levels are further enhanced, suggesting that T2DM patients may develop PAD-T2DM during the course of T2DM onset. Another study reported significantly high levels of FABP4 among female PAD-T2DM patients (8.0 ± 3.3 ng/mL) [71].

We found a weak positive correlation between FABP4 and HbA1c, FPG, BMI, total cholesterol, and LDL-cholesterol in PAD-T2DM patients. It has been previously reported that FABP4 is positively correlated with plasma triglycerides and LDL-cholesterol in T2DM, significantly influencing the lipid metabolism, and is found to be influenced by gender, age, and ethnicity [23,70,71]. FABP4 was positively correlated with BMI and body fat mass in T2DM with PAD [34]. In a case-control study with a 3-year follow-up, high FBAP4 levels were found to be significantly associated with PAD [68]. Animal model studies have indicated that FABP4 levels are essential for insulin resistance and an enhanced plasma lipid profile, thereby promoting atherosclerotic plaque formation [72]. Moreover, an association of FABP4 with atherosclerotic diseases has been reported [73,74]. Further, we analyzed the distribution of FABP4 serum levels among Nrf2 SNP genotypes. It was observed that FABP4 levels were significantly associated with Nrf2 SNP genotype TT (*p* < 0.001) and CT (*p* = 0.01) in PAD-T2DM compared to T2DM. There is no study available that reports an association between FABP4 levels and the Nrf2 SNP. Therefore, this is the first report to describe the relationship between FABP4 levels and Nrf2 SNP.

In conclusion, our study reported the association of Nrf2 SNP rs35652124 with PAD-T2DM and FABP4 levels in PAD-T2DM for the first time. Our results revealed that the genetic background plays an important role in the pathogenesis of PAD-T2DM. Therefore, a better understanding of the underlying molecular mechanisms in the context of genetic background may help improve our knowledge regarding treatment interventions and disease prevention in PAD-T2DM patients.

## Figures and Tables

**Table 1:** Clinical characteristics of the study group.

	HC	T2DM	PAD-T2DM	T2DM vs. PAD-T2DM	
	Mean ± SD			*p*-Value	95% (CI)
Age (years)	53.4 ± 7.7	58.8 ± 8.9	59.8 ± 10.6	0.6	3.3–5.3
Height (cm)	162.4 ± 4.0	165.7 ± 2.7	161.8 ± 3.8	<0.0001	2.4–5.4
Weight (kg)	59.5 ± 5.4	75.8 ± 8.7	75.6 ± 12.5	0.9	4.5–5.1
BMI (kg/m^2^)	22.6 ± 1.7	27.6 ± 3.0	28.8 ± 4.1	0.1	0.3–2.8
FPG (mg/dL)	97.2 ± 3.0	232.5 ± 56.4	259.2 ± 49.6	0.02	3.3–50.1
HbA1c (%)	5.4 ± 0.3	8.1 ± 1.7	8.7 ± 2.4	0.2	0.3–1.5
Total cholesterol (mg/dL)	185 ± 22.1	253 ± 29.1	281 ± 23.7	<0.0001	16.3–39.7
HDL-cholesterol (mg/dL)	53.1 ± 12.8	42.3 ± 7.9	39.83 ± 8.3	0.2	1.0–6.0
LDL-cholesterol (mg/dL)	122 ± 23.2	162 ± 32.1	178.1 ± 22.4	0.01	4.1–28.3
AST	18.1 ± 3.4	54.34 ± 78.1	50.8 ± 39.1	0.81	23.6–30.6
ALT	20.1 ± 4.8	32.3 ± 32.2	38.5 ± 37.4	0.4	9.0–21.5
Total bilirubin	0.58 ± 0.24	0.78 ± 0.7	1.27 ± 2.1	0.1	0.2–1.2
Serum urea	22.2 ± 7.2	52.3 ± 28.5	43.6 ± 23.3	0.1	2.7–20.1
Serum creatinine	0.74 ± 0.14	1.07 ± 0.5	1.31 ± 1.1	0.2	0.1–0.6

Abbreviations: HC—healthy control; BMI—body mass index; FPG—fasting plasma glucose; HbA1c—glycated haemoglobin; ALT—alanine transaminase; AST—aspartate transaminase; HDL—high density lipoprotein; LDL—low density lipoprotein.

**Table 2:** Distribution and frequencies of Nrf2 SNP rs35652124 genotypes and alleles.

	*n* (%)		Odds Ratio	95% (CI)	*p*-Value
	PAD-T2DM	T2DM			
Codominant					
CC	8 (19.5)	14 (34.1)	Reference		
CT	11 (26.8)	17 (41.5)	1.13	0.36–3.59	0.83
TT	22 (53.7)	10 (24.4)	3.85	1.22–12.11	0.02
Dominant					
CC	8 (19.5)	14 (34.1)	Reference		
CT + TT	33 (80.5)	27 (65.9)	2.14	0.78–5.85	0.14
Allele					
C	27 (32.9)	45 (54.9)	Reference		
T	55 (67.1)	37 (45.1)	2.48	1.31–4.67	0.005
	HC	PAD-T2DM			
Codominant					
CC	18 (43.9)	8 (19.5)	Reference		
CT	7 (17.1)	11 (26.8)	3.53	1.00–12.48	0.04
TT	16 (39.0)	22 (53.7)	3.09	1.08–8.86	0.03
Dominant					
CC	18 (43.9)	8 (19.5)	Reference		
CT + TT	23 (56.1)	33 (80.5)	3.23	1.20–8.67	0.02
Allele					
C	43 (52.4)	27 (32.9)	Reference		
T	39 (47.6)	55 (67.1)	2.25	1.19–4.23	0.01
	HC	T2DM			
Codominant					
CC	18 (43.9)	14 (34.1)	Reference		
CT	7 (17.1)	17 (41.5)	3.12	1.01–9.60	0.04
TT	16 (39.0)	10 (24.4)	0.80	0.28–2.31	0.68
Dominant					
CC	18 (43.9)	14 (34.1)	Reference		
CT + TT	23 (56.1)	27 (65.9)	1.51	0.62–3.68	0.36
Allele					
C	43 (52.4)	45 (54.9)	Reference		
T	39 (47.6)	37 (45.1)	0.91	0.49–1.67	0.75

**Table 3:** Plasma FABP4 levels among study groups.

	FABP4 Levels	*p*-Value 95% (CI)		
	Mean ± SD	HC/T2DM	HC/PAD-T2DM	T2DM/PAD-T2DM
HC	9.45 ± 3.15	<0.0001 (7.67–10.8)	<0.0001 (13.8–17.7)	<0.0001 (4.64–8.41)
T2DM	18.69 ± 3.07			
PAD-T2DM	25.22 ± 4.48			

**Table 4:** Genotypic distribution of plasma FABP4 levels among study groups.

	HC	T2DM	PAD-T2DM	T2DM/PAD-T2DM
Genotypes	FABP4 Levels (Mean ± SD)			*p*-Value
CC	9.4 ± 2.84	19.7 ± 6.95	21.7 ± 4.29	0.1
TT	9.8 ± 3.24	19.9 ± 2.07	25.7 ± 4.79	<0.001
CT	8.8 ± 2.84	19.9 ± 7.95	24.2 ± 6.56	0.01

## Data Availability

The data used to support the findings of this study are available from the corresponding author upon request.
